# Development of an Autonomous Endoscopically Implantable Submucosal Microdevice Capable of Neurostimulation in the Gastrointestinal Tract

**DOI:** 10.1155/2017/8098067

**Published:** 2017-06-21

**Authors:** J. Hajer, M. Novák

**Affiliations:** ^1^Third Faculty of Medicine, Charles University and University Hospital Královské Vinohrady, 2nd Department of Internal Medicine, Prague, Czech Republic; ^2^Faculty of Electrical Engineering, Czech Technical University in Prague, Prague, Czech Republic

## Abstract

Gastric dysmotility can be a sign of common diseases such as longstanding diabetes mellitus. It is known that the application of high-frequency low-energetic stimulation can help to effectively moderate and alleviate the symptoms of gastric dysmotility. The goal of our research was the development of a miniature, endoscopically implantable device to a submucosal pocket. The implantable device is a fully customized electronics package which was specifically designed for the purpose of experiments in the submucosa. The device was endoscopically inserted into the submucosal pocket of a pig stomach and partially severed pig side in order to adequately simulate a live animal model. The experiment confirmed that the designed device can be implanted into the submucosa and is capable of the measurement of sensor data and the transmission of this data wirelessly in real time to a computer outside of the body. After proving that the device can be implanted submucosally and transmit data, further experiments can now be performed, primarily with an electrogastrography (EGG) instrument and implantable device with tissue stimulation capability.

## 1. Introduction

Gastric dysmotility can be a sign of several relatively common diseases such as gastroparesis, which is usually characterized by chronic progression and imposes rather severe consequences on the social, work-related, and physical status of the patient. Most cases of gastroparesis are usually diabetic or idiopathic in origin and are often resistant to available medication [1]. Patients afflicted with this condition most commonly present with nausea and repeated vomiting. Based on previous research, it is known that the application of high-frequency low-energetic stimulation can help to effectively moderate and alleviate the symptoms of gastric dysmotility [2].

Based on meta-analysis, the substantial and significant improvement of symptoms and gastric emptying was observed, indicating that high-frequency gastric electrical stimulation is an effective and safe method for treating refractory gastroparesis [3]. It has also been shown that lower esophageal sphincter neurostimulator therapy is safe and effective for the treatment of GERD. There is a significant and sustained improvement in GERD symptoms, reduction in esophageal acid exposure with elimination of daily PPI usage, and no stimulation-related adverse effects [4]. However, currently, there is only one gastric-stimulation device available in the market, the Enterra II (Medtronic), which needs to be surgically implanted with the patient undergoing general anaesthesia and having a rather bulky device fitted, using intramuscular catheters which allow for stimulation of the gastric muscle tissue. This is why the option of using a wirelessly communicating device implanted into the gastric submucosal layer would represent a definite advantage and improvement in patient comfort. This would allow for a much less risky surgical procedure (in terms of general anaesthesia requirement and possible complications of both anaesthesia and surgical procedure) with a relatively simple and safe endoscopic implantation.

The goal of our research was the development of a miniature, endoscopically implantable device and its successful deployment to submucosa. This paper describes a proof of concept study which confirms that it is possible to implant a device into the submucosa which is capable to send sensor data to the receiver device outside of the body for longer periods of time. For this particular experiment, a device with a thermometer sensor, communicating using the 868 MHz industrial, scientific, and medical (ISM) license-free band, was developed and successfully tested.

This research heads towards the development of a device which could be implanted into submucosa for long periods of time (at least 5 years) and would serve as a gastric neurostimulator which could be externally programmed using a hand-held device. The purpose of the development of this novel type neurostimulator is to replace the current systems which use intramuscular leads (e.g., Medtronic Enterra II). These devices are relatively large, having a nonrechargeable battery which is placed into a subcutaneous pocket and intramuscular leads. The disadvantages of such devices are obvious—higher invasiveness (requires classical surgery with general anaesthesia) and patient discomfort. The proposed solution will not require general anaesthesia, is less invasive, and thus enhances patient's comfort.

## 2. Material and Methods

### 2.1. Implantable Device Construction

The implantable device is a fully customized electronics package which was specifically designed for the purpose of experiments in the submucosa. The device is powered by a primary Lithium Coin Cell Battery and is capable of temperature measurement and surrounding tissue stimulation using constant time and constant voltage pulses. The minimum battery life is specified to be at least 6 months. This was verified by testing the device after 6 months from new battery insertion.

### 2.2. Development of Technology for Submucosal Endoscopically Implanted Gastroneurostimulator

**Figure 1:**
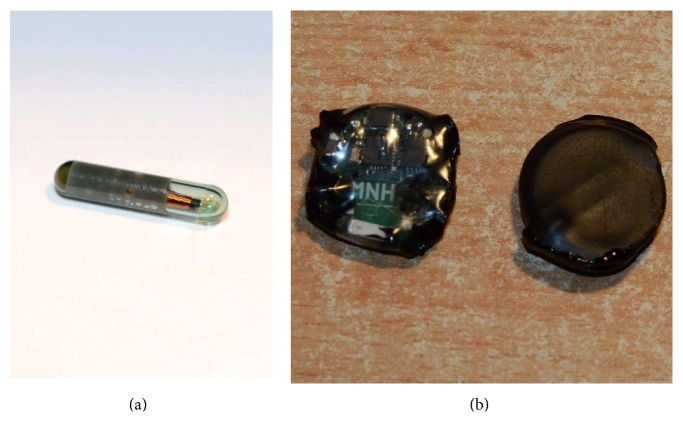
(a) 134.2 kHz LF RFID tag. (b) Encapsulated devices with sensor-only capability.

Multiple experiments were performed to determine technological aspects and construction of the device. The first experiments were done with a 134.2 kHz low-frequency radio-frequency identification (LF RFID) tag (Figure 1(a)). The tag was in the shape of a glass capsule. Inside the capsule, there is a coil with a silicon chip. The purpose of the first experiment was to confirm that a wirelessly powered object which transmits data can be endoscopically implanted into the submucosa. After implantation, the RFID tag was read using a custom-manufactured LF RFID reader with ID-innovations ID-3LA module. During the first experiment, we were able to read the RFID tag. However, due to a not ideal angular position of the implant with respect to the receiver coil (the resulting magnetic flux through the receiving coil, if the position of both coils is perpendicular to each other, is zero), we had problems with the reading of the RFID tag. We have decided to not use this design topology in future embodiments of the device and position the coil differently.

The second experiment was comprised of a battery-powered sensor which is capable of temperature measurement inside submucosa. The construction of the sensor is practically identical to the construction of the recent hardware revision. The only difference is that it does not contain conductive pads for muscle stimulation and is equipped with a different software. Polyvinylchloride and polyolefine heat-shrinkable tubes were used together with Star brite Liquid Electrical Tape to seal the implant. The completed prototypes are in Figure 1(b). The biocompatibility of the implants was not considered in this project.

### 2.3. Final Prototype Construction

#### 2.3.1. Power Management

The device is powered by a single CR2016 Energizer lithium primary cell. Its nominal voltage is 3.0 V, while the cut-off voltage is 2.0 V. The cell is cylindrical in shape and has a diameter of 20 mm and thickness of 1.6 mm. Its nominal capacity is 90 mAh under a 30 kohm load and at 21°C. The specified operating temperature range is −30°C to +60°C which is sufficient for this task where a 30°C to 40°C operating temperature is expected (implantation into ventriculus model or inside the abdominal organs of a living pig).

The LiMnO_4_ battery has a high input resistance (with respect to electronics design which draws electrical current up to 10 mA for short periods of time during transmission bursts) which changes during discharge. The value of the internal resistance varies between 15 and 100 ohms during the lifetime of the battery. The minimum required voltage for the continuous operation of the electronics without undervoltage lockout is 1.8 V. The designed circuitry involves 10 mA peak current draws which last for no more than 10 ms. Given the worst case scenario (100-ohm internal resistance), this would lead to a voltage drop of 1.0 V which would lead to an undervoltage condition. To prevent this, a 220 *μ*F ceramic capacitor is installed in parallel to the battery. It serves as an energy bank with low internal resistance to cover the energy needs connected with high current spikes.

The chips (microcontroller, RF transceiver, and temperature sensor) used do not require a stable voltage, the microcontroller and RF transmitter are capable of operation with a voltage of between 1.8 V and 3.6 V [5, 6], and the temperature sensor operates from 2.4 volts to 5.5 V [7].

Given the 90 mAh estimated capacity of the battery, a 6-month battery life requirement, and a cut-off voltage of 2.4 volts (due to the temperature sensor used), the maximum allowable current consumption of the device is 19 microamperes.

#### 2.3.2. Microprocessor Unit

The microprocessor unit handles the communication, the reading of the sensor data, and the sending of pulses for tissue stimulation. It was created using the Microchip PIC16LF1704, an 8-bit low-power microcontroller [6]. This particular microcontroller features oscillator architecture optimized for low-power devices and digital and analogue peripherals. A bit-banging SPI (Serial Peripheral Interface) bus is used for managing the communication with the radio transceiver. The analogue output from the temperature sensor is connected to a microcontroller input, configured as an analogue input. The stimulation pad outputs are configured as digital outputs. Thus, the device is capable of stimulating the muscle tissue by providing different voltage levels across the stimulation pads. The amplitude of the simulation pulse can be controlled using PWM (pulse width modulation) module inside the microcontroller. An output capacitor connected to the output of stimulation can filter out the high-frequency signal components and leave only the DC (direct-current) component of the signal. The period and duration of the stimulation pulses can be controlled using another PWM Module or Timer Module embedded in the used microcontroller. The maximum stimulation voltage and current deliverable to the tissue were, in this case, limited by the battery voltage.

#### 2.3.3. Radio Transceiver

Two options were considered for the communication circuitry. The first was inductive (also known as “near-field” communication) [8]. This method of communication is characterized by an inductive or capacitive coupling between the transmitting and receiving coils. This is particularly beneficial for power transfer. However, the particular implant presented in this paper carries a source of energy; therefore, far-field transmission was chosen. The body tissues like muscles and fat contain water which is a lossy transmission medium. The choice of operating frequency is a compromise between the attenuation of electromagnetic waves and the size of antenna. The particular material is characterized by permittivity, permeability, and conductivity.

The complex propagation constant is usually used to evaluate the effect of particular media on propagation of the electromagnetic wave [9].

(1) $$\begin{array}{c} \gamma =\sqrt{j\omega \mu \left(o+j\omega \varepsilon \right)}=\alpha +j\beta , \end{array}$$

where *γ* is complex propagation constant, *ω* is the angular frequency of the incident electromagnetic wave, *μ* is the permeability of the media, *ε* is the permittivity of the media, *α* is the attenuation constant, and *β* is the phase constant.

Using this formula, it is possible to derive the attenuation of the wave propagating through the media. For a five-layer tissue and organ model consisting of the gastrointestinal tract, visceral fat, abdomen muscle, subcutaneous fat, and skin, for three different thicknesses of the tissue from 73.1 mm to 108.6 mm, the expected loss is 17.0 to 19.2 dB for 403.5 MHz, 17.4 to 20.2 dB for 916.5 MHz, and 22.8 to 28.0 dB for 2450 MHz [9].

From this published data, the frequency in the higher sub-GHz band was chosen. The loss induced by the tissue is negligible. The link budget of the radio used exceeds 100 dB [5]. There is still a large margin for low antenna gain because of the surrounding lossy medium around it once it has been implanted. Due to radio spectrum access regulation in the European Union, a frequency of 868.0 MHz was chosen. As stated in ETSI EN 300 220-1 [10], this frequency band is dedicated to nonspecific use applications, with a maximum radiated power of 25 mW and spectrum access and mitigation of 1% or LBT+AFA (Listen Before Talk + Adaptive Frequency Agility).

The radio chipset in the device is a Texas Instruments CC110L [5]. It is a complete transceiver for ultra-low-power wireless applications. It integrates a radio baseband, modem, and simple packet engine. The clock for the transceiver is obtained by a miniature 26 MHz crystal. The differential RF port of the transceiver is connected to a Johanson Technology 0896BM15A0001 Balun [11] which matches it to a single-ended 50-ohm impedance port. A short microstrip trace leads the signal path to the matching network of the Abracon ACAJ-110-T Chip Antenna [12]. The antenna was specifically matched to operate in an area surrounded by tissue. This was done with a vector network analyzer. The ceramic chip antenna was preferred instead of a PCB (printed circuit board) antenna because the ceramic chip antennas are usually less affected by the surrounding material [13].

#### 2.3.4. Mechanical Construction and Encapsulation

**Figure 2:**
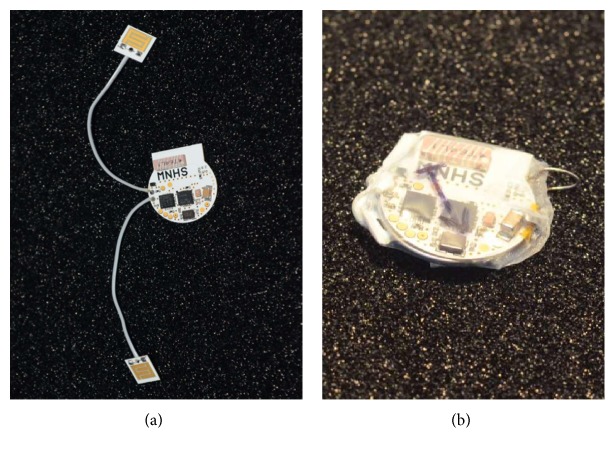
(a) Gastroneurostimulator before encapsulation. (b) Encapsulated gastroneurostimulator.

All of the electronics are manufactured on a single double-layer PCB. The thickness of the PCB is 0.3 mm and the material of the dielectrics is FR-4. All components are of SMT (surface-mount technology) and were assembled using lead-free solder paste and hot air under controlled conditions. The controlled conditions assured that the components and PCB did not go through excessive thermal stress and the maximum allowed temperature of components was not exceeded. All components are mounted on the single side except for the battery which is mounted on the reverse side. The contact wires for the electric stimulator functionality wires were soldered to the solder pads on the PCB. The other side of the wires is soldered to gold-plated contact plates manufactured using the same technology as the main PCB. The complete sensor before encapsulation is in Figure 2(a).

To ensure sufficient hermeticity for the purpose of the initial experiments with the device, the printed circuit board was coated with a conformal coating. Then, the device is encapsulated into either a polyvinylchloride or polyolefin heat-shrinkable tube. To seal the edges, the procedure differs based on the type of shrinkable tube. For a PET tube, one of the ends is sealed with heat while the other, which has the leads to stimulating plates, is filled with a waterproof and elastic silicone adhesive and sealant. For a polyolefin heat-shrinkable tube, the silicone sealant is used for both sides. The biocompatibility of the implants was not considered in this project. After curing, all devices were tested by placing them into a 50 cm high column of saturated salt solution with water. This test has proven that the devices are sufficiently waterproof and that they do not suffer from any major leaks. Any major water leak into the device would increase the current draw from the battery which would be easily detectable by the electronics as a sudden drop of the battery voltage. The encapsulated device is in Figure 2(b).

#### 2.3.5. Receiver Hardware and Software

The receiver side is supported by a Texas Instruments TrxEB evaluation and development board for radio transceivers. The board was used together with SmartRF Studio 7 software for PC and was connected to a PC via a USB interface. A module with CC110L transceiver was inserted into the development board. The module features an SMA connector. A quarter monopole antenna tuned for 868 MHz operation is used.

**Figure 3:**
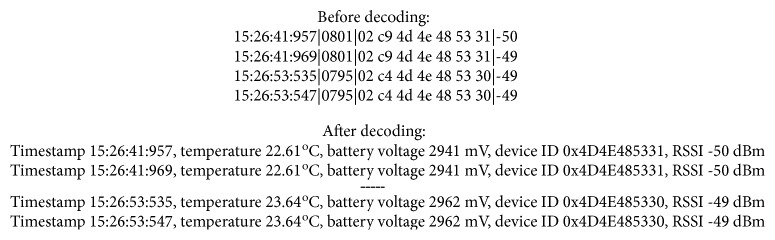
Raw data received from the implant before and after decoding; device 1 has ID 0x4D4E485330, while device 2 has ID 0x4D4E485331.

The device transmitted a 9-byte payload approximately every 22 seconds. A simple protection against burst interference by sending two packets immediately one after another was implemented. The first 2 characters of the payload represent the analogue temperature reading, followed by the battery voltage and serial number (which was chosen randomly). The packets were decoded using a script programmed in Python (Figure 3()).

One device was implanted into the stomach (device 1) while the other (device 2) was placed on the table.

### 2.4. Animal Models

We used an animal model for endoscopy (using a fresh, flushed-out pig stomach with the adherent oesophagus and duodenum). The model used is a standard endoscopic one commonly used for the training of techniques such as ESD (endoscopic submucosal dissection), tunnelisation, and POEM (peroral endoscopic myotomy). In order to visualize the contact of the device with the external reader, the model stomach with the device implanted was isolated with the use of a pig's side in order to simulate a live experimental animal model as closely as possible.

### 2.5. Endoscopic Insertion and Attachment of Device

**Figure 4:**
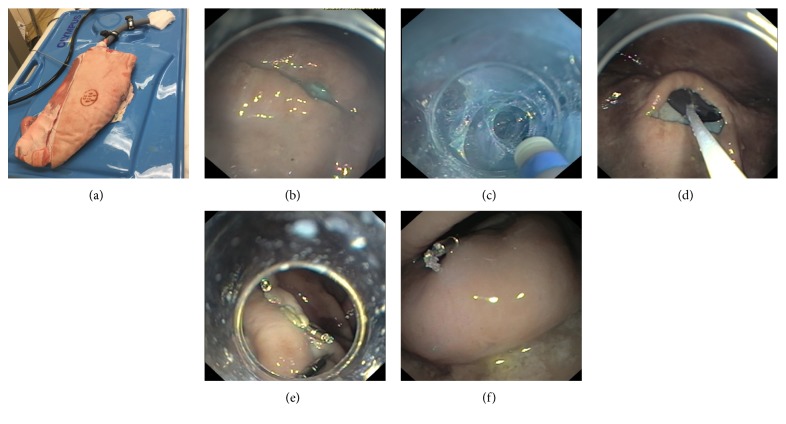
View of device implantation and endoscopic technique. (a) Animal model Olympus. (b) Submucosal incision (an entrance for the submucosal pocket formation). (c) Tunnelisation of the submucosal pocket using the Dual Knife. (d) Device implantation. (e) Closing the entry with haemostatic clips. (f) The implanted device inside the submucosal pocket, located in close proximity to the antral muscle.

In order to perform the implantation and visualization, we used an animal-model-dedicated endoscope (Olympus GIF-Q 160), which was inserted in the standard way into the animal model consisting of a pig stomach and side pork in order to adequately simulate a live animal model. The device was grasped externally with a mesh (Twister Plus, Boston Scientific) and then inserted into the stomach, where it was released. The endoscope was then extracted, equipped with a dissection cap (15.5 mm FujiFilm lens Hood), and then inserted into the stomach again. In order to implant the device into the submucosal layer in the pyloric antrum, a technique first described by Deb et al., called an endoscopic submucosal pocket (ESP), was used [14]. Following the application of a saline solution combined with methylene blue into the submucosal layer using an injection therapy needle catheter (Boston Scientific, 25G), a horizontal incision was made in order to create an opening in the submucosa using a Dual Knife (Olympus; electrosurgical knife with knob-shaped tip). Using the affixed cap (15.5 mm FujiFilm lens Hood), we inserted the cap into the newly created space, and with the use of a Dual Knife, we continued disrupting, dilating, and dissecting the submucosal layer, creating a sufficiently large-enough pocket to implant the stimulation device. The device, lying freely inside the stomach, was grasped by the insertion and extraction loops and, using a grasper (Olympus Alligator Jaw grasping forceps), navigated into the submucosal pocket, placed in close proximity of the muscularis propria. Haemostatic clips (Instinct endoscopic hemoclip, Boston Scientific) were used to secure the device in place inside the submucosal pocket and prevent any mispositioning or dislodging (Figure 4).

## 3. Results

An endoscopic placement of the gastric neurostimulator was successfully performed in all device dimensions (12 mm × 3 mm, 25 × 22 × 5 mm, and 25 × 22 × 6 mm). The weight of the second development prototype with sensor-only functionality is 3.8 grams. The final prototype with stimulation capability was further mechanically optimized; its weight is 3.1 grams. In order to implant the device into the submucosal layer, we used a technique first described by Deb et al., called an endoscopic submucosal pocket (ESP). We attached the stimulator near the muscular layer (muscularis propria) where it is theoretically the optimal stimulation depth. Creating the submucosal pocket and implanting of the gastric neurostimulator endoscopically took 20–30 minutes. During this procedure, there is no intraprocedural complication such as perforation. As our study was performed on an animal model, the risk of bleeding, infection, or device migration associated with this method is unknown. Based on experience with endoscopic submucosal dissection, endoscopic tunnel dissection, and endoscopic suturing, these potential risks are expected to be minimal.

The battery life of the device exceeds 6 months. This was tested using a prototype which was not implanted. After 6 months from insertion of battery, the capability of the device to transmit data was tested using the TrxEB board. There was no degradation in performance in terms of link stability and received signal strength. The voltage was measured both remotely by decoding the sensor data and destructively by opening the sealed sensor and measuring the voltage directly to confirm. The battery voltage was 2.55 volts, which means that there was still some battery capacity left.

The one-directional communication link from the device to receiver was successfully tested. The results show that the performance of 868 MHz link was not affected by the presence of the tissue. Two identical devices were used in the experiment. The distance from both devices to the antenna receiver was about 50 cm. The received signal strength from both devices is very similar. The fact that the received signal strength from the device covered by tissue is so similar to the received strength of the device placed on the table can be explained by the fact that the chip antenna was matched to its characteristic impedance in the presence of tissue to achieve the best performance. The antenna surrounded only by air is then mismatched, which results in lower radiated energy.

The device is capable of neurostimulation. The maximum voltage of the stimulation is limited by the battery voltage of the current prototype. In determining the shape of stimulation pulses (on-time, off-time frequency of stimulation pulses and length of pulses), we have reviewed the datasheet of Medtronic Enterra II gastric neurostimulator [15]. The pulse length is 60 to 450 microseconds, on time and off time is 0.1 seconds to 24 hours, and pulse frequency ranges from 2 Hz to 130 Hz. The prototype device is capable of reproducing the parameters mentioned above, except for the maximum stimulation voltage.

## 4. Discussion

If the lumen was historically the first space and the peritoneal cavity was the second, then the intramural space has come to represent the “third space.” Unlike the others, this space remains virtual and has to be created by dissecting and expanding the tissue layer between the mucosa and the muscularis propria, allowing the endoscope to gain access [16]. Currently, submucosal techniques represent a new challenge for therapeutic endoscopists. Considering our past experiences with these techniques, we decided to implant the stimulation device directly into the submucosa, using the endoscopic submucosal pocket technique [14]. This particular method is proven to be relatively safe, simple, and minimally invasive. The average procedure time was 25 minutes, which we consider to be the minimum amount of time needed in order to correctly place the device into the submucosa and position it so that it remains in close contact with the gastric muscles. However, for the following experiments on live animal models, it will probably be necessary to modify our approach and avoid using haemostatic clips, which only close the mucosal opening and could later lead to implant rejection. In this case, it would be advantageous to use over the scope (OVESCO) clips instead, seeing as these would allow us to close the entry across all three of the stomach layers, including the muscle layer. Confirming these methods and techniques will require follow-up experiments on live animal survival models, which will also confirm the safety of a long-term submucosal implantation of the stimulation device.

From a technical point of view, the limiting parameter of the implantable solutions to the submucosa is the overall size. For its simplicity, a battery-only solution was developed. However, the battery makes up a great portion of the device, limiting the functionality and/or constraining the size of the device. The design of the wireless communication part, on the other hand, was relatively straightforward due to the nature of the radio receivers which feature extremely high sensitivity (beyond −100 dBm) and channel selectivity [5]. From a broader perspective, the research and development is heading toward two other sensor topologies—a battery-less variant powered from outside of the body and a rechargeable battery which is capable of holding its charge for between a few days to a few weeks with the ability to be wirelessly recharged. This would make the device capable of autonomous operation which is beneficial mainly in terms of exposure of human tissue to electromagnetic field (which is linked to specific absorption rate (SAR)) where battery-less solutions require continuous energy transmission while battery-powered solutions do not.

The major technological challenges in the realization of a fully compliant gastric neurostimulator can be divided into four main groups—encapsulation, communication, power transfer, and securing the implant in place. The device is expected to stay in the submucosa for very long periods of time, typically months to years. There are several parts of the implantable device which have to be taken into account—the main package, inputs and outputs, and lead connections [17]. Given the very small size of the submucosal implantable device, all of these aspects have to be carefully taken into account to avoid any sealing failure. The current hardware revision of the neurostimulator which features nonrechargeable battery could benefit from a metallic enclosure which usually offers better hermetic sealing than polymer, glass, or ceramic materials [18]. A combination of metal material for encapsulation of critical subsystems (electronic components) and glass/ceramic material for the whole device would be a good trade-off between the influencing of wireless energy transfer or communication and the hermeticity of the device. Two-way communication between the implant and a device outside the body can be realized via a UHF wireless link, preferably in MEDS frequency band (402–405 MHz) intended for use with implantable medical devices. The high sensitivity of today's radio frequency receivers can compensate for the increased path loss in then lossy medium (the tissue). The battery life of the current prototypes is limited to several months. Changing the neurostimulator several times a year would be very impractical. Wireless charging could solve this issue. Inductive power transfer is a well-studied subject with commercially available applications (e.g., wireless charging of smartphones) [19]. Wireless power transmission is an intensively studied subject. Papers show that wireless transfer of small amounts of power to a medical implant is feasible even over distances of around 20 cm [20]. Two approaches for securing the implant are proposed. These approaches should secure the implant for long periods of time and avoid unwanted travel inside submucosa. The first is to use a biocompatible synthetic scaffold as a coating for the device. The surrounding tissue should grow into the scaffold, securing it in place. The second possible solution is more radical and involves securing the implant in place using an OVESCO clip. The implant could be attached to the OVESCO clip used to close the submucosal pocket after implanting, which would limit possible implant travel in the tissue. Both approaches should also limit any rotation of the implant. The feasibility of both approaches is a subject of further research. The synthetic scaffold could also solve the problem with implant rejection which is likely to happen if the enclosure for the implant is not manufactured from biocompatible material.

## 5. Conclusions

The final experiment confirmed that a device which is endoscopically implantable can be used for the stimulation of muscle tissue in the gastrointestinal tract. Despite being tricky and not very straightforward to implement due to a very lossy propagation of radio waves through tissue, bidirectional communication using the 868 MHz ISM band was successfully established. In the future, further experiments will be performed, primarily with an EGG instrument and an implantable device with tissue stimulation capability. The maximum stimulation voltage is planned to be 12 V. This will be achieved by using a step-up DC-DC converter which will generate the required voltage during stimulation. For communication, MedRadio (Medical Device Radiocommunications Service) band will be used to evaluate the feasibility of realization of the device which would be fully compliant to legislation in terms of the frequency used, allowed output power, and mitigation. The development of a reliable and ultra-low-power bidirectional communication link is the key to the realization of a system which could be controlled and checked from outside of the body by the user using a standard personal device (smartphone, tablet, computer, etc.).

The research is heading towards a smaller solution which will be powered externally, will be capable of staying in the submucosa for years without the need for maintenance, and will be able to receive external commands with information of how to stimulate the tissue. The goal of this paper was to show a way of implementing and implanting a gastric neurostimulator into the submucosa. Submucosal implants appear to be an interesting form of treatment of a broad spectrum of gastrointestinal diseases such as gastroesophageal reflux, incontinence, and sphincter dysfunctions.
